# A Combination of Systemic and Intracranial Anti-CD25 Immunotherapy Elicits a Long-Time Survival in Murine Model of Glioma

**DOI:** 10.1155/2009/963037

**Published:** 2010-03-21

**Authors:** Marie-Denise Poirier, Houda Haban, Abdeljabar El Andaloussi

**Affiliations:** Laboratory of Immuno-Oncology and Tumor Immunotherapy, Immunology Program, Faculty of Medicine and Health Sciences, University of Sherbrooke, Sherbrooke, QC, Canada J1H 5N4

## Abstract

Abrogating the suppression of glioma-infiltrating Tregs in the periphery and the central nervous system is essential to successful glioma rejection. We sought to improve the immune response in glioma-bearing mice, by investigating new strategies using the anti-CD25 immunotherapy. We found a complete long-term survival of glioma-bearing mice treated with a combination of systemic and intracranial anti-CD25 mAb immunotherapy as compared to systemic administration of anti-CD25 mAb. In addition, the group of mice that had been cured by the combined anti-CD25 mAb showed long-term survival without late tumor relapse when challenged with the GL261 glioma. The antitumor immune response was investigated by analysis of antitumor immune response (CTL). Results showed that the use of the combined injections of anti-CD25 mAb induced efficient targeting of Tregs expansion inside and outside of the brain and altered Tregs trafficking in the bone marrow and brain areas where antitumor immunity was primed.

## 1. Introduction

Glioblastoma tumors are characterized by extensive microvascular infiltration and rapid proliferation. Targeting tumor infiltrating lymphocytes (TIL) as well as regulatory T cells (Tregs) can completely destroy established solid tumors by favoring antitumor immune responses. An active suppression by Tregs plays an important role in the downregulation of T cell responses to foreign and self-antigens in the peripheral immune system. However, convincing data regarding the role of Tregs in tumors of the central nervous system (CNS) have been accumulating only during the last few years. Efforts aimed at developing new therapies have focused on strategies that specifically target tumor cells while sparing normal cells. One such approach, immunotherapy, has shown promises within the spectrum of agents used against malignant brain tumors. 

Tumor-infiltrating lymphocytes (TILs) have been found and characterized in glioma and in several experimental models [1–3]. The presence of TILs indicated that they were involved in the induction of a local immune response but this response was not sufficient to control or reject the tumor because of the suppressive effect of Tregs [4]. Tregs are described as CD4^+^CD25^+^ T cells that often coexpress cytotoxic T lymphocyte-associated protein 4 (CTLA-4) [5], glucocorticoid-induced tumor necrosis factor receptor (GITR) [6], lymphocyte activation gene-3 (LAG-3), CD28 [7], OX-40 [8], and 4-1 BB [9]. Of significance, Tregs constitutively express the forkhead family transcription factor Foxp3 [10, 11]. In addition, Tregs express a series of markers such as CD62L, CD69, neuropilin-1, and the Th2 chemokine receptor 4 (CCR4) in a relatively specific manner [12, 13]. 

The constitutive expression of CD25 on the surface of Tregs has allowed the use of anti-CD25 monoclonal antibodies for depletion studies. Recently, Tregs depletion using anti-CD25 mAb by injection intracranially has resulted in a gain of survival of mice bearing an established glioma as well as an enhancement of CD8^+^ T cell frequency [1]. The anti-CD25 mAb is directed against the Tac epitope of the CD25 molecule, to which it binds without leading to complement fixation, antibody-mediated cellular cytotoxicity or relevant CD25 modulation [14]. It has been reported that CD25 can also be a potent activator of Tregs in vivo and in vitro [15]. 

In the present study, we showed for the first time that the treatment of glioma-bearing mice by systemic and intratumoral injections of anti-CD25 mAb induced complete rejection of glioma in a murine model. These results contrasted with those of a systemic anti-CD25 treatment alone in which case the partial depletion of Tregs was not sufficient to cure all of the treated mice [1]. 

## 2. Materials and methods

### 2.1. Tumor Cell Line and Animals

GL261 glioma cells were obtained from American Type Culture Collection (Manassas, VA). The cells were cultured in Dulbecco's Modified Eagle's Medium (DMEM) supplemented with 10% fetal calf serum, 5 mM L-glutamine, streptomycin (100 *μ*g/mL), and penicillin (100 units/mL) at 37°C in a humidified atmosphere containing 5% CO_2_. C57BL/6 female mice (6–8 weeks old) were obtained from Harlan (Indianapolis, IN). The mice were housed and maintained under pathogen-free conditions in accordance with a protocol approved by the Institutional Animal Care and Use Committee of the University of Sherbrooke.

### 2.2. Tumor Models

Mice were anesthetized with an intraperitoneal injection of 0.1 mL of a stock solution containing ketamine hydrochloride 25 mg/mL, xylazine 2.5 mg/mL, and 14.25% ethyl alcohol diluted 1  :  3 in 0.9% NaCl. For stereotactic intracranial injections of tumor cells, the surgical site was shaved and prepared with 70% ethyl alcohol and Prepodyne solution. After a midline incision, a 1 mm right parietal burr hole centered 2 mm posterior to the coronal suture and 2 mm lateral to the sagittal suture was made. Animals were then placed in a stereotactic frame and 1 × 10^5^ GL261 tumor cells were injected using a 26-gauge needle to a depth of 3 mm over a period of 3 minutes. The total volume of injected cells was 5 *μ*L. The needle was removed, the site was irrigated with sterile 0.9% NaCl, and the skin was sutured with 4.0 nylon.

### 2.3. Intracellular Cytokine Staining

Cells were stained using anti-CD8 (clone 53-6.7), anti-CD4 (clone L3T4), and anti-CD25 (clone 3C7) mAbs purchased from BD Bioscience (Mississauga, ON), followed by fixation. The cells were then permeabilized (*BD* Cytofix/Cytoperm, BD Biosciences) and stained with anti-INF-*γ*, anti-IL4 (clone 11B11), and anti-Foxp3 (clone FJK-16s) mAbs (eBioscience, San Diego, CA). Intracellular Foxp3 was stained using the protocol suggested by the supplier. 

### 2.4. CTL

Splenocytes (1 × 10^6^ cells) were washed and stained using antimouse CD8 (clone 53-6.7), CD62L (clone MEL-14), CD44 (clone P2A1) (BD Bioscience), and CD107a mAbs. The cells were washed, and three-color flow cytometry analysis was performed using a FACSCalibur cytometer (Becton Dickinson). Cells in the lymphocyte gate staining positively for CD8*α* were analyzed for CD62L and CD44 expression [16], using the Flow-Jo software (Becton Dickinson).

### 2.5. Experimental Groups

There was a total of three experimental groups in the case of the in vivo studies (*n* = 10 mice/group). One group consisted of mice injected intracranially with GL261 alone as described above and previously without anti-CD25 treatment [1]. A second group comprised mice injected intracranially with GL261 and treated systemically by injection of anti-CD25 mAb (0.1 mL at 100 ng/*μ*L). A third group corresponded to mice injected intracranially with GL261 and treated by systemic (0.1 mL at 50 ng/*μ*L) and intracranial (0.1 mL at 50 ng/*μ*L) injections of anti-CD25 mAb. The injections began one week after tumor implantation. The mice were given three injections per week for three weeks. All experiments were performed in triplicates (*n* = 3).

### 2.6. Statistical Analysis

Statistical comparisons of the level of expression of different markers used for Tregs characterization in the various experimental groups were performed using the Student paired *t*-test. Survival was plotted using a Kaplan-Meier survival curve and statistical significance was determined by the Kruskal-Wallis nonparametric analysis of variance followed by the nonparametric analog of the Newman-Keuls multiple comparison tests. A *P*-value of  .05 or less was considered significant. 

## 3. Results

### 3.1. The Progression of Glioma Accompanied Bone Marrow Tregs Expansion

**Figure 1:**
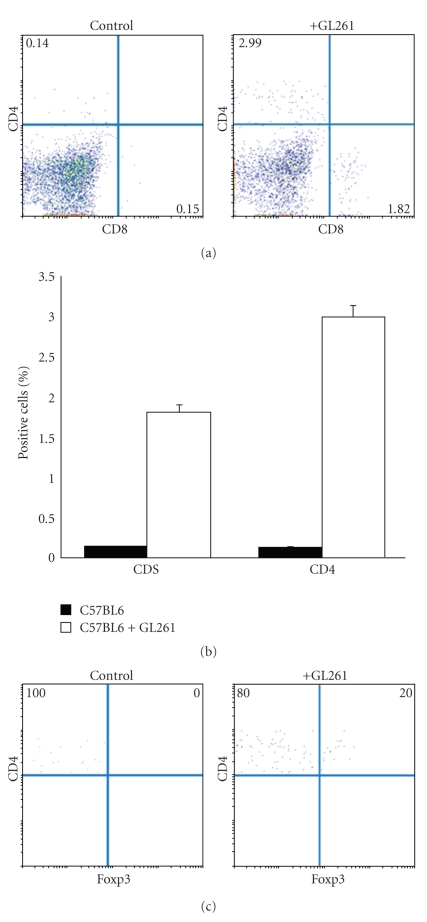
FACS analysis of CD4^+^, CD8^+^, and CD4^+^Foxp3^+^ T cells subsets in the bone marrow of glioma-bearing mice. (a), (b) The frequency of CD4^+^ and CD8^+^ T cells was significantly increased (*P* < .002) in the bone marrow of glioma-bearing mice as compared to control (glioma-free) mice. (c) Intracellular staining of Foxp3 in gated CD4^+^ T cells from the bone marrow of glioma-bearing and glioma-free mice. Data are shown as the percentage average of positive cells and represent the mean of three independent experiments with three mice per group (*P* < .002).

**Figure 2:**
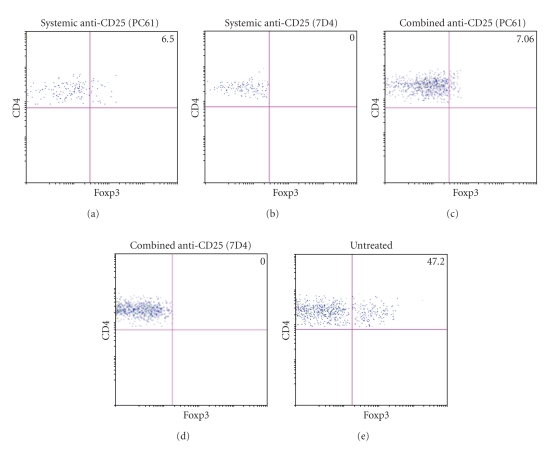
Depletion of Tregs by anti-CD25 mAb clone PC61 or anti-CD25 mAb clone 7D4. (a) Tregs infiltration in untreated glioma-bearing (control) mice. (b), (c) Tregs depletion in mice treated by systemic or combined injections of the anti-CD25 mAb PC61. (c), (e) Tregs depletion in mice treated by systemic or combined injections of the anti-CD25 mAb 7D4. Data are presented as the percentage average of positive cells and represent the mean of three independent experiments (*P* < .001).

We analyzed the effect of the glioma on the expansion of the populations of CD4^+^ and CD8^+^ T cells as well as Tregs in the bone marrow of glioma-bearing mice at three weeks after tumor implantation (three mice per group). The FACS analysis revealed an increase in the percentage of CD4^+^ (2.99 ± 0.15) and CD8^+^ (1.82 ± 0.13) T cells in the bone marrow of glioma-bearing mice when compared to untreated glioma-free mice (controls). Results in control animals were 0.14 ± 0.06 (CD4^+^) and 0.15 ± 0.04 (CD8^+^) (*P* < .002) (Figures 1(a) and 1(b)). The intracellular expression of Foxp3 in gated CD4^+^ T cells from the bone marrow of glioma-bearing mice confirmed the presence of Tregs in 20.0% ± 2.33% of the cells. In contrast, there was an absence of these cells in control mice (Figure 1(c)). Based on these findings as well as the fact that the bone marrow represents the immediate tumor environment of glioma, bone marrow-residing Tregs expressed higher levels of Foxp3 (Figure 2(a)) as compared to control mice (Figure 1(c)).

### 3.2. The Combined Systemic and Intracranial Treatments with Anti-CD25 mAb Cured Glioma-Bearing Mice

**Figure 3:**
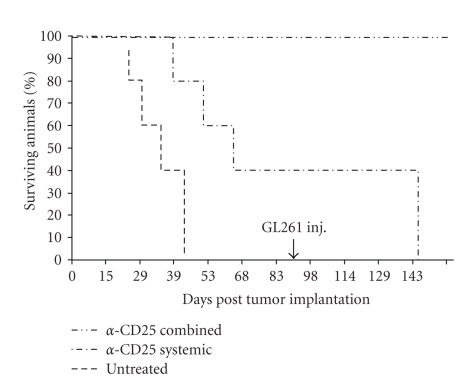
Combined anti-CD25 immunotherapy prolongs the survival of glioma-bearing mice. Kaplan-Meier survival graph showing a significant increase in the median length of survival in mice (*n* = 10) treated with systemic and intratumoral anti-CD25 mAb (*P* < .005) and rechallenged with GL261.

We tested the efficiency of two anti-CD25 mAbs clones (PC61 and 7D4) to deplete Tregs in glioma-bearing mice as well as their capacity to induce tumor rejection in these animals. Control animals were untreated glioma-bearing mice. Results of the combined systemic and intracranial treatments revealed that the brain of the glioma-bearing mice showed no evidence of tumor growth in long-term survivors, independently of the anti-CD25 mAb tested. FACS analysis of TILs based on intracellular Foxp3 staining showed a complete depletion of Tregs in animals that were treated with the combined anti-CD25 immunotherapy (Figures 2(c) and 2(d)). In the case of mice treated by systemic injections of anti-CD25 mAbs alone, results showed similar percentage values whether mAb PC61 (6.5% ± 0.45%) or mAb 7D4 (7.06% ± 0.6%) was used (Figures 2(a) and 2(b)), in contrast to the untreated glioma-bearing mice (47.2% ± 3.2%) (*P* < .001) (Figure 2(e)). All the mice bearing intracranial GL261 tumors that were treated with the combined anti-CD25 mAb immunotherapy remained alive (100% survival) over the period of observation (150 days). In contrast, 40% of the mice that received only the systemic injection of anti-CD25 remained alive over the same period of time. All the untreated glioma-bearing animals were dead 43 days following implantation of the tumor (Figure 3). We investigated the efficiency of each regimen of anti-CD25 immunotherapy (systemic and combined) to prevent late tumor relapse. Anti-CD25-treated surviving mice were challenged with GL261 tumor cells 95 days after the first implantation and left untreated. All the mice that had previously been treated by systemic anti-CD25 administration were dead 49 days later whereas the animals that had previously been treated by combined anti-CD25 administration remained alive, with apparent healthy conditions (Figure 3).

### 3.3. The Administration of Anti-CD25 mAb Modified the CD4^+^/CD8^+^ T Cells Ratio in Brain and Bone Marrow

**Figure 4:**
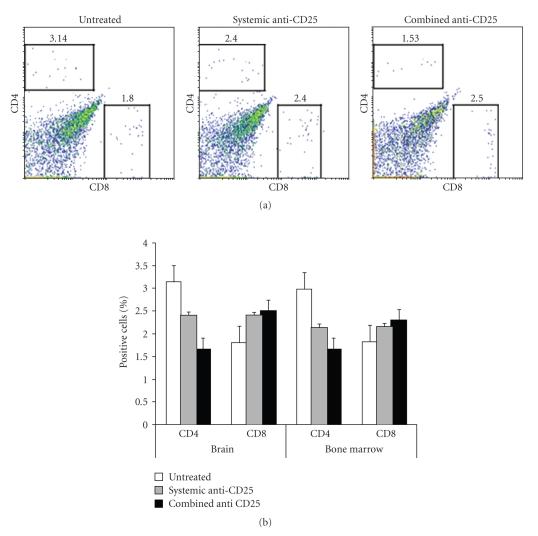
Effect of anti-CD25 mAb immunotherapy on TILs of glioma-bearing mice. (a) FACS dot plot of TILs analyzed by CD4 and CD8 double staining. (b) Distribution of CD4^+^ and CD8^+^ T cells within context of anti-CD25 glioma immunotherapy, compared between brain and bone marrow. Data are presented as the percentage average of cells and represent the mean of three individual experiments (*P* < .001).

The ratio of TIL CD4^+^ and CD8^+^ T cells in the brain as well as bone marrow was evaluated three weeks after tumor implantation in untreated, systemic-treated, and combined-treated glioma-bearing mice to assess the effect of Tregs depletion on the expansion of CD8^+^ T cells infiltrating the glioma. The TIL cell suspension was stained with anti-CD4 and anti-CD8 mAbs. FACS analysis revealed an inversion of the CD4/CD8 ratio in untreated glioma-bearing mice, in which case data were 3.14 ± 0.4 (CD4^+^)/1.80 ± 0.07 (CD8^+^), giving a ratio of 1.74. In the case of mice treated with the combined anti-CD25 mAb immunotherapy, results were 1.53 ± 0.02 (CD4^+^)/2.5 ± 0.05 (CD8^+^), giving a ratio of 0.52. In the instance of the group of mice treated by systemic injection of anti-CD25 mAb, the results were 2.4 ± 0.06 (CD4^+^)/2.4 ± 0.05 (CD8^+^), giving a ratio equal to one (Figure 4(a)). Data were significantly different between the three sets of data (*P* < .001). In addition, the same effect on the ratios of CD4^+^/CD8^+^ T cells was observed in the bone marrow (Figure 4(b)). The ratios were 1.64 in the case of untreated mice (2.99 ± 0.32 (CD4^+^)/1.82 ± 0.1 (CD8^+^)), 0.72 in the instance of the combined immunotherapy (1.66 ± 0.17 (CD4^+^)/2.3 ± 0.25 (CD8^+^)), and 1.06 in systemic-treated mice (2.13 ± 0.15 (CD4^+^)/2.15 ± 0.35 (CD8^+^)). Differences between the three sets of data were significant (*P* < .001).

### 3.4. The Combined Immunotherapy Treatment Induced the Generation of CD8^+^CD62L^low^CD44^high^ CTLs

**Figure 5:**
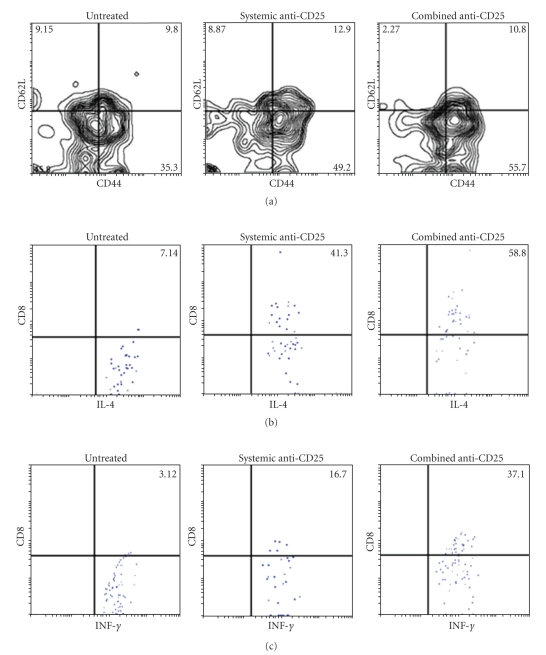
Phenotype of CD8^+^ CTL induced by anti-CD25 combined immunotherapy. (a) Dot plots of FACS analysis of triple staining for expression of CD8, CD44, and CD62L, after gating the CD8^+^ T cell population in comparison with untreated glioma-bearing mice. (b) Cytokine intracellular staining of IL-4 and INF-*γ* (c) versus CD8. The mean fluorescence intensity of each marker is given in each dot plot. Values represent the mean fluorescence intensity of three independent experiments ± SD.

Tregs accumulate inside tumors and act to maintain a local cytokine environment that suppresses the effector function of tumor-infiltrating CD8^+^ T cells [17]. Systemic administration of anti-CD25 mAb has previously been shown to deplete Tregs in tumor models and autoimmune disease [18]. Here, we employed a novel strategy which made use of two different routes (systemic and systemic/intracranial) of administration of anti-CD25 mAb. The phenotype of CTL CD8^+^ T cells infiltrating the brain of glioma-bearing mice treated with anti-CD25 was assessed by FACS analysis using the cell surface markers CD62L and CD44. Results showed that the CTLs were CD8^+^CD62L^low^CD44^high^ T cells. Furthermore, results revealed a frequency of CTL in mice treated with the combined anti-CD25 immunotherapy (55.7% ± 3.5%) that was higher than that in systemic (49.2% ± 3.1%) or untreated (35.3% ± 2.6%) animals (*P* < .001) (Figure 5(a)).

### 3.5. The Immune Response Targeted the Glioma: Role of Interferon Gamma and IL-4

The expression of INF-*γ* and IL-4 cytokines was examined in CTL by intracellular staining and FACS analysis. The results of CD8^+^ T cells infiltrating the brain were compared in the cases of untreated, systemic-treated, and combined immunotherapy-treated glioma-bearing mice. The CTL population induced as a result of anti-CD25 immunotherapy expressed high levels of INF-*γ* and IL-4 in the group of glioma-bearing mice that had been treated, as compared to untreated animals (Figure 5). In the case of INF-*γ*, results were 37.1% ± 2.1% (combined immunotherapy), 16.7% ± 1.5% (systemic immunotherapy), and 3.12% ± 0.7% (absence of treatment). Data for intracellular staining of IL-4 were 58.8% ± 2.8% (combined immunotherapy), 41.3% ± 2.4% (systemic immunotherapy), and 7.14% ± 1.2% (absence of treatment). There was a significant difference between the three sets of data, with reference to the absence of treatment (*P* < .002) (Figures 5(b) and 5(c)). The cytotoxic capacity of CD8^+^ T cells was also examined through the expression of CD107a (lysosomal-associated membrane protein 1), which is described as surrogate marker for cytolytic activity. Indeed, the frequency of CD107a positive cells was substantially higher in TILs of combined treated mice when compared to untreated and systemic immunotherapy-treated glioma-bearing mice (data not shown).

## 4. Discussion

Abrogating the suppression of glioma-infiltrating Tregs in the periphery and the central nervous system is essential for a successful rejection of glioma. Recent studies have shown that Tregs suppressed the activation/proliferation of CD4^+^ or CD8^+^ T cells in an antigen-specific manner [4, 19]. We have previously reported that the infiltration of gliomas by Tregs correlated with the stage of progression [20]. Therefore, the current experiments were designed to test the hypothesis that Tregs exert highly immunosuppressive effects and allow rapid growth of malignant brain tumors. In the present study, we provide evidence for the efficiency of a novel and promising strategy that combines the use of intracranial and systemic administrations of anti-CD25 mAb in an experimental glioma murine model. 

Data on the use of anti-CD25 mAb monoimmunotherapy in models of glioma are accumulating. However, this approach has yet to be shown to induce efficient tumor rejection and long-term survival in murine models of glioma. Furthermore, fundamental questions remain on the most efficient way to use this treatment, reflecting the complexity of the brain immune system. The rationale basis for the use of anti-CD25 is that the mAb inhibits clonal proliferation of autoreactive CD4^+^ T cells and Tregs function by blocking the binding of IL-2 to its receptor [1, 21, 22]. The systemic administration of anti-CD25 has been shown to prolong the survival of glioma-bearing mice by functional inactivation and partial depletion of glioma-infiltrating Tregs [1, 22]. The partial depletion of Foxp3^+^ Tregs in the murine glioma model may depend on the nature of the mAb used. For instance, treatment of mice using the anti-CD25 mAb clone PC61 has been reported to partially deplete Tregs whereas the anti-CD25 mAb clone 7D4 did not [23, 24]. In contrast, we showed here that targeting Tregs by combined systemic and intracranial injections of anti-CD25 mAb eradicated glioma from the brain of glioma-bearing mice, conferred long-term survival, and prevented relapse, whether anti-CD25 mAb clone PC61 or 7D4 was used. These observations stressed the importance of the strategy of immunotherapy as opposed to the nature of the anti-CD25 mAb in the murine model of glioma.

There are a few reports concerning the number and function of immunosuppressive Tregs in the bone marrow. This is particularly surprising for two reasons. First, the bone marrow represents a major priming site for T cell responses and second, Tregs play important roles in promoting tumor escape from T-cell-dependent immunosurveillance [25, 26]. In this connection, we have reported that the infiltration of the bone marrow of glioma-bearing mice by Tregs correlated with the progression of tumor growth in the brain of a murine model as well as in glioma patients [20]. Here, we found an increase in the population of Tregs in the bone marrow of glioma-bearing mice whereas there was an absence of Tregs in the case of tumor-free (control) mice and no change in the percentage of Tregs as well as the ratio of CD4^+^/CD8^+^ in secondary lymphoid organs as spleen, neck, and cervical lymph node (data not shown). Our observations supported the interpretation that the bone marrow represented the immediate tumor environment of glioma. Based on this observation, we compared the effects of systemic and combined (systemic and intratumoral) anti-CD25 mAb treatments on bone marrow-residing Tregs in glioma-bearing mice. The results showed a complete depletion of Tregs from the bone marrow of mice treated with the combined anti-CD25 mAb immunotherapy in contrast to systemic anti-CD25 immunotherapy and untreated animals. These observations confirmed that the major expansion of Tregs from CD4^+^CD25^−^ T cells occurred in the bone marrow of glioma-bearing mice. Furthermore, our data are in agreement with the work of Fecci et al. [22] who suggested that an increase of Tregs in the bone marrow is the result of a selective trafficking that could be in part from the thymus, where we have reported an increase of Tregs in untreated glioma-bearing mice [27], and other secondary lymphoid organs. The Tregs become in the bone marrow before raising the glioma tumor in the brain of the mice. The conversion and/or expansion of Tregs in the bone marrow of glioma-bearing mice are not clear and require further investigations. 

Tregs can suppress not only CD4^+^ T cells, but also CD8^+^ T cells and B cells [28]. Many observations indicate that tumors expressing costimulatory molecules are usually rejected more efficiently in vivo [29]. A role of costimulatory signals on peripheral tumors may be to enhance and/or maintain primed T cell responses against the tumor [30, 31]. Specific CTLs are present in the brain of glioma-bearing mice treated with anti-CD25 and they can be activated by a suitable immunization protocol. Fecci et al. [22] have reported that systemic anti-CD25 mAb enhanced T cell proliferation and INF-*γ* production and strengthen antigen-specific anti-glioma CTL response. In agreement with this finding, we observed that the antitumor effects of Tregs depletion were mediated by a T-cell-dependent antitumor immune response. The efficient effect of combined anti-CD25 strategy contributed to the induction of a CD8^+^CD62L^low^CD44^high^ CTL response directed against GL261. 

In summary, we have shown that the combined systemic and intratumoral injection of anti-CD25 mAb resulted in powerful antitumoral effects that were revealed by tumor rejection, complete survival, and resistance to relapse. Our data suggest the existence of physiologic tumor-targeted immune response in the central nervous system mediated by Treg depletion from the bone marrow of glioma-bearing mice. The strategy and results reported here may be used as a basis for a more effective anti-CD25 immunotherapy in glioma patients.
